# Mitochondrial Functioning and Its Relation to Higher-Order Cognitive Processes †

**DOI:** 10.3390/jintelligence8020014

**Published:** 2020-04-01

**Authors:** Alexander P. Burgoyne, Randall W. Engle

**Affiliations:** School of Psychology, Georgia Institute of Technology, 654 Cherry St., Atlanta, GA 30332, USA; burgoyn4@gmail.com

One of the most replicated findings in psychology is the *positive manifold* between cognitive ability measures (Jensen 1998; Spearman 1904). That is, scores on different tests of cognitive ability are positively correlated, implying the existence of a *g* factor. Our research attempts to explain these relationships among broad cognitive abilities using a combination of experimental and differential approaches (Cronbach 1957).

Nearly twenty years ago, one of us suggested that *attention control*, reflecting the ability to maintain goal-relevant information and inhibit interference, might account for the relationship between working memory capacity and fluid intelligence (Engle 2002). Evidence from experimental work and individual difference studies converged on the idea that attention control was the common thread, connecting performance on tests of working memory, which required maintenance of goal-relevant information, and tests of fluid intelligence, which required disengagement from no-longer-relevant information (Engle 2018).

Recent developments have allowed us to improve the measurement of our ability to control our attention—to keep it from being captured by either external events or internal thoughts and emotions. These new measures have demonstrated higher reliability estimates, stronger inter-correlations, and higher loadings on a common factor than traditional measures of attention control (Draheim et al. forthcoming). We have also shown that attention control, when measured properly, mediates the relationship between fluid intelligence and working memory capacity (Draheim et al. forthcoming), mediates the relationship between sensory discrimination ability and fluid intelligence/working memory capacity (Tsukahara et al. forthcoming), and adds substantial incremental validity to the Armed Services Vocational Aptitude Battery used by the American military to make job assignments (Martin et al. forthcoming). Taken together, this work suggests that one explanation for the positive manifold may be differences in ability to control attention. Even so, we do not see attention control as the one single explanation for differences in intelligence.

Naturally, when we saw Geary’s (2018) article, “Efficiency of Mitochondrial Functioning as the Fundamental Biological Mechanism of General Intelligence (*g*)”, we wondered to what extent mitochondrial functioning might underpin individual differences in attention control. If mitochondrial functioning imposes a “ceiling on the performance of higher-level systems” as Geary (2019, p. 3) suggests, it might explain why some people are better able to selectively focus their attention than others, or “ramp up” the intensity of their attentional focus. Consistent with this possibility, Geary (2019) suggests that mitochondrial functioning may be related to the ability to “[maintain] focus during a novel and complex problem-solving task” (p. 3).

However, an explanation of fluid intelligence as at least partially due to differences in attention control only goes so far. A central issue in all of scientific psychology is there is always a deeper level of analysis. Our theory begs the question “So, why do people differ in ability to control attention?” Of course, we can make informed speculations about brain mechanisms, and even less-informed speculations about genetic and epigenetic factors, but even these beg the question “So, what factors allow these variables to implement differences in behavior that would be defined as intelligent?” Geary’s theory is an educated guess at an even deeper level of analysis, and there is much to recommend it.

Our view is that Geary’s theory is complementary to our own work, providing a biologically plausible explanation at a lower level of analysis for the shared variance among broad cognitive abilities. Further, the idea encourages us to move away from the anthropocentric view that we are only talking about differences in human intelligence. It is growing ever clearer that the ideas of working memory capacity, fluid intelligence, and attention control are important individual difference variables for nonhuman animals as well, even mice and rats (Matzel et al. 2013).

At first glance, however, our concern was that Geary (2018, 2019) was proposing a “theory of everything”, promising to unify disparate research areas and providing a single explanatory mechanism for a wide range of phenomena, including *g*, health, and aging. Our concerns were at least two-fold: first, that single-variable explanations rarely (if ever) fully account for the functioning of complex systems, and second, that by cutting across levels of analysis, but situating the explanatory mechanism squarely outside most cognitive scientists’ area of expertise, the theory would be exceedingly difficult to test, at least by people who study and theorize about intelligence and cognition.

Geary (2018, 2019) has undertaken an admirable effort to unify disparate fields, but makes no claim that the efficiency of mitochondrial functioning is the one true cause—solely responsible for the phenomena he links it to, nor that it accounts for most of the variance. In fact, most (if not all) of Geary’s claims are hedged and testable; he even notes the patterns of results that would refute them (Geary 2019, p. 1030).

The merits of Geary’s theory are that it is generative and falsifiable. It is *generative*, in that it evokes specific predictions about relations between fundamental biological and higher-order processes. These predictions will undoubtedly motivate researchers to work together towards new frontiers and expand on the suggestive evidence Geary presents. It is *falsifiable*, in that it makes testable claims about *g*, cardiovascular health, and aging. It therefore lends itself to a number of different testbeds, and consequently, avenues for falsification.

That said, as is the case with any psychological theory, falsification requires the appropriate research design, and this is one issue we think Geary could resolve with clarification. Specifically, we were left wondering how large a sample would be needed to detect significant relations between mitochondrial efficiency and higher-order cognitive processes, such as attention control, working memory capacity, and fluid intelligence. As is well known, underpowered studies are susceptible to *Type II errors* (failure to reject the null when it is false). In turn, Type II errors can lead researchers to conclude that a theory is wrong, simply because they lacked the power to properly test it. 

By way of example, genome-wide association studies have shown that massive samples (tens of thousands of participants, or more) are needed to detect significant effects of single-nucleotide polymorphisms on intelligence, because they typically account for tiny fractions of the total variance. Although Geary (2018, 2019) argues that mitochondrial efficiency is a piece of the puzzle, he does not provide an estimate of “how big” a piece of the puzzle it is. Mixed evidence from the mtDNA polymorphism studies he references, however, appears to suggest that very high-powered studies will be required to test his claims using correlational designs.

Perhaps the most controversial claim Geary (2019) makes is that “Cellular energy is the lowest common currency driving the development and expression of all biological systems and thus places upper-limit constraints on the development and expression of all other systems” (p. 2). Along similar lines, he states, “*The key question* is whether variation in mitochondrial functioning is the *most fundamental* underlying biological mechanism of *g*” (Geary 2018, p. 1036, italics added). Importantly, however, he notes that a number of sub-processes contribute to the efficiency of mitochondrial functioning, including “ATP production, immunological functions, inflammation, control of oxidative stress, and intracellular signaling” (Geary 2018, p. 1041). Each of these subprocesses can be examined “under the microscope”, and, presumably, each can be attributed to its own set of precursors and subprocesses. Our point is that Geary’s (2018) “key question” invites methodological reductionists to dig deeper, until the tools currently available can go no further. Geary’s level of analysis seems approachable by comparison.

One wonders to what extent such a micro-level explanation will resonate with cognitive scientists, and how it might inform higher levels of analysis. From a historical perspective, this reductionist approach seems reminiscent of the discovery of subatomic particles (neutrons, protons, quarks, leptons, and so on). One consequence is that fewer people with increasingly specialized knowledge are able to contribute to the discussion, and fewer still are able to connect it to the bigger picture. This is not to dissuade researchers from pursuing this level of analysis, as surely we should seek to understand everything we can about human and nonhuman animal functioning. Rather, our argument is that, at least historically, the question of “why” something occurs is never truly resolved, as there is always another “why?” lurking around the corner. Good scientists often play the role of a 3-year-old child, continually asking “Why?” to every answer.

Finally, one might predict a diminishing role for psychologists moving forward, if explanations of behavioral phenomena become more and more molecular. As counterpoint, researchers interested in explaining these phenomena via lower levels of analysis are constrained by the behaviors elicited by tasks participants are asked to perform. Designing and testing these cognitive tasks to ensure they are valid and reliable for their intended purpose is work that falls clearly in the realm of differential and experimental psychologists. Moreover, the process of designing and testing tasks is critically important to identifying specific cognitive processes and components necessary to understand intelligence in its full complexity. Thus, one contribution we will continue to make as psychologists is investigating the components of these tasks, and further refining them through psychometrics. 

Ultimately, Geary’s theory is ambitious, and we are eager to see where it leads the field. Work in this area will require collaboration with researchers who do not often cross paths. We view this as a potential merit, but presenting a clear barrier to entry, too.
